# Cardiovascular risk profiles clusters among children and adolescents with disabilities

**DOI:** 10.1186/s12889-023-15796-4

**Published:** 2023-05-16

**Authors:** Maayan Bar Yehuda, Sharon Barak, Yeshayahu Hutzler, Kwok Ng, Ariela Giladi, Lilach Bar Meir, Adilson Marques, Avi Zigdon, Moti Zwilling, Orna Reges, Yossi Harel Fisch, Riki Tesler

**Affiliations:** 1 Health Promotion Research Center, Ariel University, Ari’el, Israel; 2 Department of Nursing, Faculty of Health Science, Ariel University, Ari’el, Israel; 3 Levinsky-Wingate Academic Center, Wingate Campus, Netanya, Israel; 4 Physical Activity for Health Research Cluster, Department of Physical Education and Sport Sciences, University of Limerick, Limerick, Ireland; 5 School of Educational Sciences and Psychology, University of Eastern Finland, Joensuu, Finland; 6 Faculty of Education, University of Turku, Rauma, Finland; 7 School of Education, Bar Ilan University, Ramat Gan, Israel; 8 CIPER, Faculdade de Motricidade Humana, Universidade de Lisboa, 1499-002 Lisbon, Portugal; 9 Department of Economics & Business Administration, Faculty of Social Sciences, Ariel University, Ari’el, Israel; 10 Department of Health System Management, Faculty of Health Science, Ariel University, Ari’el, Israel

**Keywords:** Cardiovascular Diseases, Risk Profiles, Adolescents, Disabilities

## Abstract

**Background:**

Cardiovascular diseases (CVD) are a precursor for disabilities and death worldwide. Being overweight or obese in combination with physical inactivity and smoking habits may increase the risk for CVD and other health problems such as lower limb osteoarthritis, diabetes, stroke, and various cancer types among children and adolescents. The literature emphasizes the need to follow such groups and evaluate the risk of individuals developing CVD diseases. Therefore, the current study explores the variety of cardiovascular risks in children and adolescents’ profiles clusters with and without disabilities.

**Methods:**

Data from 42 countries including Israel, was collected with the support of the world health organization (WHO, Europe) through a questionnaire from 11–19 years old school-aged.

**Results:**

The study finding shows that children and adolescents with disabilities demonstrated a higher prevalence of overweight than those who completed the HBSC youth behavior survey. Moreover, the prevalence of tobacco smoking and alcohol use was statisticaly significantly higher among the disabled group than the non-disabled group. In addition, socioeconomic status of responders who presented a very high CVD risk was found as significantly lower than those from the first and second low risk groups.

**Conclusion:**

This led to the conclusion that children and adolescents with disability were at a higher risk of developing CVDs than their non-disabled peers. In addition, intervention programs tailored to the needs of adolescents with disability should consider lifestyle habit change and promoting healthy living thus improving their quality of life as well as reducing their risk of being exposed to severe CVD diseases.

## Background

Cardiovascular diseases (CVDs) are the leading cause of premature disability and death wordwide [1]. The total prevalence of CVD cases doubled from 271 million in 1990 to 523 million in 2019 and the number of CVD deaths increased from 12.1 million in 1990, reaching 18.6 million in 2019. Likewise, the number of years that people who live with a disability due to CVDs doubled from 17.7 million to 34.4 million over the same period [2]. Years lived with disability due to CVDs doubled from 17.7 million to 34.4 million from 1990 to 2019 [2]. An estimated 17.9 million people died from CVDs in 2019, representing 32% of all global deaths [3]

The most prevalent risk factors leading to the onset of CVDs are being overweight or obese, engaging in low levels of physical activity, smoking, and alcohol use [4]. The prevalence of being overweight has increased over the past five decades [5, 6]. The World Health Organization has declared that the increased prevalence of being overweight or obese can be attributed to the reduction of physical activity, as well as the sedentary lifestyle adopted by most of the world's population, and to the increased consumption of fatty high-calorie food [7, 8]. Moreover, recent studies emphasize the association between overweight and obesity with increased risk for serious health problems, such as osteoarthritis of the lower limb joints, type 2 diabetes, stroke, coronary heart disease, and various types of cancers [2, 9–13].

### Physical activity

Physical activity is known to reduce the risk for the development of cardiometabolic diseases, depression, and various types of cancers, as well as improve blood pressure and bone density [13, 14]. On the other hand, physical inactivity is associated with a higher risk of being overweight or obese, as well as leading to cardiorespiratory impairment [14]. Thus, developing a physically active lifestyle at an early age may decrease the risk of developing health problems, such as obesity, diabetes, and CVDs [15].

### Smoking

Research has shown that cigarette smoking causes diseases in nearly all body organs [16]. Smoking has been well established as a risk factor for developing CVD, including coronary heart disease and peripheral arterial disease, abdominal aortic aneurysms, ischemic nephropathy, bowel ischemia, aortic dissection, cancer and stroke [17]. Global tobacco, including primary smoking, secondhand smoke, and use of chewing tobacco, accounted for 8.71 million deaths and 230 million disabilities in 2019; 36.7% of the death cases were due to CVDs [2]. Research has shown that cigarette smoking usually begins before 18 [16]. Cigarettes, cigars, smokeless tobacco, e-cigarettes, hookahs, pipe tobacco, and bidis are the most common types of tobacco use among students in grades 6–12 [18]. In 2015, 4.7 million middle and high school students were estimated to be using some type of tobacco product [19].

### Alcohol abuse

Epidemiologic studies in the last two decades have demonstrated strong associations between alcohol use and cardiovascular conditions such as hypertension, coronary heart disease, stroke, peripheral arterial disease, and cardiomyopathy [20]. Excessive use of alcohol has been found to increase the disability-adjusted life years in ischemic heart disease [2]. The global prevalence of alcoholic cardiomyopathy in 2019 was estimated to be 708,000 cases (approximately 9.1 cases per 100,000). Alcoholic cardiomyopathy was found to be responsible for 71,700 deaths in 2019 [2]. Interestingly, the correlation between alcohol use and CVDs is modulated by the dose and pattern of alcohol consumption. Low-to-moderate daily alcohol consumption (15 to 20 g/day; 1 to 2 standard drinks) has been associated with a reduced risk of CVDs and mortality, while higher amounts of alcohol consumption have been linked to increased risk [20].

### Adolescents with disabilities

Disability can be dfined as a long-term physical, mental, intellectual or sensory impairments which in interaction with various barriers may hinder their full and effective participation in society on an equal basis with others [21]. The disability is usually associated with a decrease in the quality of life and increased use of various health and social services. According to the Centers for Disease Control and Prevention (CDC) 1 out of 6 children aged 3–17 (17.3%) have special health care needs and that 61 million adults in the United States live with a disability [22–27]. Not only that, 6.7% of them have two or more developmental disabilities [23].

A meta-analysis study that explored physical activity among youth with disabilities (including 729 participants, 4–20 years old) revealed that the time spent in moderate to vigorous physical activity decreases with age, whereas time spent in light physical activity stays the same as they age [14]. Across European countries, approximately 15% of girls and 23% of boys with disabilities took part in daily moderate to vigorous physical activity [24].

In addition, adolescents with emotional disabilities, learning disabilities, and mobility impairments were found to be more likely to experience health risks compared to those with no disabilities [25]. Blum et al., performed a survey among 20,780 12^th^ grade youth with mobility impairments, learning disabilities, and emotional disabilities and found out that this population was statisticaly significantly more likely to report regular smoking habits than youth without such disabilities in the same age range [25].

The current study was aimed to build specific clusters of modifiable health behaviours across adolescents with and without disabilities and further to explore the cardiovascular risk profiles among adolescents with disabilities in those clusters. We hypothesized that adolescents with disability exhibit lifestyle characteristics that may contribute to the development of CVDs.

## Methods

### Data collection

Data for this study were obtained from the Health Behaviour in School-aged Children (HBSC) study, a cross-national survey with support from the World Health Organization (WHO, Europe), which was completed in 2018/2019 in 42 countries, including 40 European countries, Canada, and Israel.

The current study included adolescents aged 11 to 19 years old from the Israeli HBSC study. Sampling was conducted following the structure of the national education system. The primary sampling unit was the school class. The whole school served as the primary sampling unit if a sample class was not available. If a school with two or more classes was selected, the one chosen for the sample was randomly selected.

Data were collected using self-reported standardized questionnaires, which were administrated in the school classrooms. Students did not provide personal details (such as name, classroom, teacher), making them completely anonymous and ensuring their confidentiality. Researchers strictly followed the standardized international research protocol to ensure consistency in survey instruments, data collection, and processing procedures. Response rates at the school, class, and student levels exceeded 80%.

### Measures

#### Sociodemographic information

The HBSC consists of items describing the children’s sociodemographic characteristics, including their self-identified sex (boy or girl), date of birth, and socioeconomic status using the Family Affluence Scale III (FAS). The FAS is a 6-item questionnaire regarding material assets in the home, measured based on the number of vehicles owned, bedroom sharing, computer ownership, number of bathrooms at home, dishwashers at home, and family vacations [26]. The scale has a total score that ranges from 0 (lowest) to 17 (highest). The FAS is a better proxy of socioeconomic status than measures that rely on adolescent reports of parental occupation or income [27].

#### Disability status

Self-reported functional difficulties were used to generate a marker for disabilities based on the Washington Group on Disability Statistics [28]. The HBSC is solely based on self-report by adolescents; therefore, the Washington Group on Disability items were slightly modified to reflect this. More specifically, the terminology in the original questions was modified from proxy items to self-report. For example, the proxy question “Does [child’s name] have difficulties in...” was changed to “Do you have difficulties in... ”.” At the time of data preparation. At the time of data preparation, the UNICEF child functioning module was not fully developed [29]. The short six sets were used for this population with the following functions: seeing, hearing, walking, remembering, learning, and concentrating. Each question had a four-point response scale to describe difficulty, including: “Not difficult at all”, “A little difficult”, “Very difficult”, and “I cannot do it at all”. Answers were dichotomized into “Disability” (“I cannot do it at all” or “Very difficult”) and “No-disability” (“Not difficult at all” or “A little difficult”). According to Ng et al., two macro functioning groups were formed by grouping the cognitive functions (remembering, learning, concentrating) as well as the physiological functions together (seeing, hearing, walking)), with acceptable test–retest reliability [30].

#### Risks for developing cardiovascular diseases

Four different types of modifiable lifestyle characteristics that have been proven to contribute to the development of CVDs were examined: overweight and obesity, physical activity level, weekly tobacco smoking, and weekly alcohol consumption.

#### Overweight and obesity

Bodyweight status was assessed using body mass index (BMI) and interpreted according to the World Health Organization (2020) cut-off values in five categories: underweight (< -2 standard deviations), normal weight (-2 standard devations to + 1 standard deviation), overweight (> + 1 standard deviation), and obese and very obese (> + 2 standard deviations). Following, data were dichotomized into those who were “Normal weight” and into those who were “Overweight or obese’ [13]

#### Physical activity level

According to a previously published study, physical activity was defined by “any activity that usually increases your heart rate and makes you get out of breath some of the time”. Accordingly, physical activity level was assessed using the question, that first had a definition of intensities of physical activity and some examples and then the question “How often have you been physically active over the past seven days for a total of at least 60 min per day?” Answers were given on an 8-point scale (0 = none to 7 = daily). The measure has reasonable validity (*r* = 0.37) with 5-day accelerometer data and acceptable test–retest reliability when used as a dichotomous variable. (intraclass correlation coefficient range: range 0.694–0.765) [31, 32].

At the time of data collection, the World Health Organization recommended for sufficient physical activity for children and adolescents (5–17 years old) at least 60 min of moderate intensity physical activity daily [33]. This recommendation has since changed from daily to 60 min a day on average. Through a slight change, we still kept to the old recommendation and participants' responses were converted into the following two categories: Not daily (6 or fewer active days) and daily (7 active days) based on the World Health Organization (2010) recommendations. the prevalence for each group was calculated.

#### Tobacco weekly smoking habits and alcohol use

The HBSC questionnaire includes questions investigating tobacco use and alcohol consumption. The current study defined weekly tobacco smoking habits based on the question ‘‘How often do you smoke tobacco at present?’’ Original answer categories (never, less than weekly, weekly but not daily, daily) were recoded into "Weekly smoking" and "Less than weekly smoking". Additional questions ask how often individuals drank beer, wine, and liquor/spirits. For each type, response options were ‘‘1 = never’’,’’2 = rarely’’, ‘‘3 = every month’’, ‘‘4 = every week’’, ‘‘5 = every day’’. This variable was dichotomized by combining options 1 through 3 (indicating less than weekly alcohol use, and 4 and 5 (to reflect at least weekly alcohol use [34].

### Statistical analysis

#### Descriptive statistics

Descriptive statistics (means, standard deviations, sample sizes, and percentages) were used to describe the sociodemographic and lifestyle risks for CVDs (overweight and obesity, physical activity level, tobacco smoking habits, and alcohol use) of the entire sample, students without disability, and students with disability. In addition, chi-square tests were used to compare the lifestyle of participants without and with disabilities. Finally, independent t-tests (continuous variables) and chi-square tests (categorical variables) were used to compare the demographic characteristics of the study’s participants and those who were omitted from the analyses on account of missing data.

#### Cluster analysis

Identifying clusters of modifiable risk factors allows for the design of health-oriented intervention health promotion programs during adolescents’ formative years. Accordingly, using cluster analysis, we aimed to analyze adolescents with and without disabilities profiles according to their modifiable lifestyle CVD risk factors. More specifically, four variables assessing four different modifiable lifestyle cardiovascular risk factors were included in the cluster analysis: obesity (overweight/obese vs. normal/under weight), physical activity level (daily vs. not daily physical activity), tobacco smoking habits (weekly vs. less than weekly smoker) and alcohl use (weekly vs. less than weekly consumer). The type of cluster analysis conducted was a two-step cluster analysis. This type of analysis is a hybrid approach that first uses a distance measure to separate groups and then a probabilistic approach to choose the optimal subgroup model [35, 36]. This type of cluster analysis has numerous advantages compared to more traditional techniques, such as determining the number of clusters based on a statistical measure of fit rather than on an arbitrary choice, using categorical and continuous variables simultaneously, analyzing outliers, and being able to handle large datasets [36, 37]. Two-step cluster analysis is considered reliable in terms of the number of subgroups detected, classification probability of individuals to subgroups, and reproducibility of findings on clinical and other types of data [36].

In the first step (pre-clustering), a sequential approach was used to pre-cluster the cases based on the definition of dense regions in the analyzed attribute space. Following, the pre-clusters were statistically merged in a stepwise manner until all clusters were in one cluster [38]. The cut-off for favourable lifestyle behaviour was set at 20%, meaning ≤ 20% of the sample presented unfavourable lifestyle behaviour. Following, based on the number of unfavourable risk factors in each cluster, clusters were labelled as “low risk” (one unfavourable behavior), “moderate risk” (2 unfavorable behaviors), “high risk” (3 unfavorable behaviors), and “very high risk” (4 unfavorable behavior).

Finally, sociodemographic characteristics (age, socioeconomic status, sex, and disability status) of the clusters were evaluated and compared. The continuous variables (age and socioeconomic status) were evaluated using a one-way analysis of variance with the Tukey–Kramer post-hoc test. Normal distribution was also evaluated using the D’Agostino-Pearson test. Categorical variables were evaluated using the chi-squared test.

In all analyses, the level of significance was set to *p* < 0.05 (two-tailed) using IBM SPSS Statistics (version 23.0).

## Results

### Sociodemographic characteristics of the study group and missing data

The total study population (*n* = 3,900) was composed of 56.79% females and 43.20% males (mean age: 14.52 ± 2.07 years; range: 11–19 years). A total of 78.28% (*n* = 3,053) of the sample (*n* = 3,053) did not have any disability. Participants without disability socioeconomic status was higher than this f participants with disabilities (*p* < 0.05) (Table 1). Participants were recruited from Jewish (63.07% of the sample) and non-Jewish (36.92%) schools. A total of 507 individuals were not included in the analyses on missing data on a healthy lifestyle. In comparison to those who were not included in the analysis, youth who were included in the analysis were statisticaly significantly older (13.63 ± 2.12 years vs. 14.52 ± 2.07 years, respectively; *p* < 0.05) than use not included in the analyses. Moreover, compared to youth not included in the analyses, the socioeconomic status of those included in the analyses was statisticaly significantly higher (FAS = 8.08 ± 2.69 vs. 8.48 ± 2.51, respectively; *p* < 0.05). No additional between-group differences were observed (Appendix).

**Table 1:** Participants’ demographic characteristics (*n* = 3,900)

Variables	Total sample (*n* = 3900) N (%)	No disability (*n* = 3053) N (%)	Disability (*n* = 847) N (%)	Only physiological disability (*n* = 292) N (%)	Only cognitive disability (*n* = 210) N (%)	Physiological and cognitive disability (*n* = 345) N (%)
Age, years; mean (SD) [range]	14.52 (2.07) [11.00–19.00]	14.49 (2.09) [9.00–19.00]	14.57 (2.01) [11.00–18.00]	14.54 (2.01) [11.00–18.00]	14.67 (1.98) [11.00–18.00]	14.50 (2.06) [11.00–19.00]
Sex, n (%)—Males	1685 (43.20)	1303 (42.67)	382 (45.10)	132 (45.20)	98 (46.66)	152 (44.05)
Socioeconomic status (Family Affluence Scale), mean (SD) [range]^b^	8.48 (2.51) [1.00–17.00]	8.64 (2.45) [1.00–17.00]	7.94 (2.65)^a^ [1.00–17.00]	7.87 (2.71)^a^ [1.00–17.00]	7.97 (2.65)^a^ [1.00–17.00]	8.00 (2.60)^a^ (1.00–17.00]

^a^Statisticaly significantly different from “No disability group” (*p* < 0.05, 2-tailed); ^b^ The Family Affluence Scale is a 6-item questionnaire. For the purpose of this study, a total score was calculated. Total score ranges from 0 (lowest) to 17 (highest); SD, standard deviation

### Modifiable lifestyle characteristics

Although most of the study participants had a healthy weight, approximately 21.1% of the sample was overweight or obese. In terms of physical activity level, 9.0% of the sample took part in daily physical activity. In terms of smoking and alcohol use, a large proportion of adolscents reported that they do not engage in weekly smoking or alcohol use (91.1% and 90.1%, respectively).

The healthy lifestyle characteristic of the non-disabled group (*n* = 3053) and the disabled group (*n* = 847) were compared. The results indicated that in comparison to the non-disabled group, in the disabled group, the prevalence of obesity (19.91% vs. 25.8%, respectively, *p* < 0.05), tobacco smoking (6.84% vs. 12.9%, respectively, *p* < 0.05), and alcohol use (8.09% vs. 12.9%, respectively, *p* < 0.05), were statistically significantly higher. In the next step, participants with disability were grouped into those with only physiological disability (*n* = 292), only cognitive disability (*n* = 210), and those with both physiological and cognitive disability (*n* = 345). In most between-disability group comparisons, no statistically significant differences were found. Accordingly, the entire disability group was used without examining each disability group separately in all further analyses (Table 2).

**Table 2:** Population lifestyle characteristics (*n* = 3,900)

Variables		Total sample (*n* = 3900) N (%)	No disability (*n* = 3053) N (%)	Disability (*n* = 847) N (%)	Only physiological disability (*n* = 292) N (%)	Only cognitive disability (*n* = 210) N (%)	Physiological and cognitive disability (*n* = 345) N (%)
Overweight or obese	No	3079 (78.9)	2445 (80.08)	628 (74.2)^a^	196 (67.12)^b,c^	191 (90.95)^a^	298 (86.37)^a^
	Yes	821 (21.1)	608 (19.91)	219 (25.8)^a^	96 (32.88)	19 (9.05)	47 (13.63)
Moderate to vigorous physical activity	Daily	351 (9.0)	274 (8.97)	66 (7.8)	14 (4.79)	17 (8.09)^c^	13 (3.76)^b^
	Not Daily	3549 (91.0)	2779 (91.02)	781 (92.2)	278 (95.21)	193 (91.91)	332 (96.24)
Tobacco smoking	Less than weekly	3551 (91.1)	2844 (93.15)	738 (87.1)^a^	267 (91.43)^b^	205 (97.61)^a^	326 (94.49)
	Weekly	349 (8.9)	209 (6.84)	109 (12.9)^a^	25 (8.56)	5 (2.39)	19 (5.51)
Alcohol use	Less than weekly	3513 (90.1)	2806 (91.90)	738 (87.1)^a^	276 (94.52)	201 (95.71)	334 (96.81)
	At least weekly	387 (9.9)	247 (8.09)	109 (12.9)^a^	16 (5.48)	9 (4.29)	11 (3.19)

^a^Statisticaly significantly different from “No disability group” (*p* < 0.05, 2-tailed); a, statistically significantly different from “Only physiological disability” group (*p* < 0.05, 2-tailed); b, statistically significantly different from “Only cognitive disability" group (*p* < 0.05, 2-tailed); c, statistically significantly different from "Physiological and cognitive disability" group (*p* < 0.05, 2-tailed)

### Cluster analysis

#### Types of clusters

Results from the two-step cluster analysis led to a five-cluster classification as the optimal solution for the data considered in the present study. Following a parsimony criterion, the five-cluster solution presented the greatest ratio distance measure (1.45), which is based on the current number of clusters against the previous number of clusters.

According to the risk stratification labelling described in the statistical analysis section, there were two low-risk clusters: the first low-risk cluster (*n* = 2424, 62.15% of the sample) and the second low-risk cluster (*n* = 351, 9% of the sample). The third cluster (*n* = 613, 15.71% of the sample) presented a moderate risk for CVDs. The fourth cluster (*n* = 203, 5.20% of the sample) was composed of children presenting a high risk for CVDs. Finally, the fifth cluster presented very high risk for CVDs (*n* = 309, 7.92% of the sample).

#### Sociodemographic characteristics of the five clusters

Significant between-clusters differences were found in age. More specifically, participants in cluster 2 (low risk – group 2) were statsiticaly significantly younger than in the other 4 clusters. Moreover, the socioeconomic status of cluster 5 (very high risk) was statisticaly significantly lower than that of clusters 1, 2 (low-risk groups 1 and 2), and 4 (high-risk group; Table 3).

**Table 3:** Description of the five clusters of cardiovascular risk according to lifestyle behavioral characteristics and demographic characteristics (*n* = 3,900)

Variables		Cluster 1: LCR-1 (*n* = 2424)	Cluster 2: LCR-2 (*n* = 351)	Cluster 3: MCR (*n* = 613)	Cluster 4: HCR (*n* = 203)	Cluster 5: VHCR (*n* = 309)	Chi-square test (*p* value) OR *F*-value (*p* value)
Overweight or obese, n (%)	No	2424 (100.0)^b,c,d^	277 (78.9)^a,c,e^	0 (0.0)^a,b,d,e^	156 (76.8)^a,c^	222 (71.8)^a,b,c^	2955 (< 0.0001)
	Yes	0 (0)	74 (21.1)	613 (100.0)	47 (23.2)	87 (28.2)	
Moderate to vigorous physical activity, n (%)	Daily	0 (0.0)^b^	351 (100.0)^a,c,d,e^	0 (0.0)	0 (0.0)	0 (0.0)	3900 (< 0.0001)
	Not daily	2424 (100.0)	0 (0.0)	613 (100.0)	203 (100.0)	309 (100.0)	
Tobacco smoking, n (%)	Less than weekly	2424 (100)^b,e^	311 (88.6)^a,c,d,e^	613 (100.0)^b,d,e^	203 (100.0)^b,e^	0 (0.0)^a,b,c,d^	3465 (< 0.0001)
	Weekly	0 (0.0)	40 (11.4)	0 (0.0)	0 (0.0)	309 (100.0)	
Alcohol use, n (%)	Less than weekly	2424 (100.0)^b,d,e^	308 (87.7)^a,c,d,e^	613 (100.0)^b,d,e^	0 (0.0)^a,b,c,e^	168 (54.4)^a,,c,d^	3900 (< 0.0001)
	At least weekly	0 (0)	43 (12.3)	0 (0.0)	203 (100.0)	141 (45.6)	
Age, in years, mean (SD)		14.60 (2.07)^b,d^	13.60 (2.03)^a,c,d,e^	14.35 (2.05)^b,d^	15.32 (1.80)^a,b,c,e^	14.73 (1.99)^b,d^	28.07 (< 0.0001)
Socioeconomic status, mean (SD)		8.51 (2.46)^b,c,d,e^	8.98 (2.53)^a,c,e^	8.12 (2.53)^a,b,d^	9.13 (2.46)^a,c,e^	8.00 (2.72)^a,b,d^	12.99 (< 0.001)

*Abbreviations*: *LCR-1* Low cardiovascular risk (group 1), *LCR-2* Low cardiovascular risk (group 2), *MCR* Moderate cardiovascular risk, *HCR* High cardiovascular risk, *VHCR* Very high cardiovascular risk

^a^Statisticaly significantly different from “Cluster 1” (*p* < 0.05, 2-tailed)

^b^Statisticaly significantly different from “Cluster 2” (*p* < 0.05, 2-tailed)

^c^Statisticaly significantly different from “Cluster 3” (*p* < 0.05; 2-tailed)

^d^Statisticaly significantly different from “Cluster 4” (*p* < 0.05, 2-tailed)

^e^Statisticaly significantly different from “Cluster 5” (*p* < 0.05, 2-tailed)

**Fig. 1:**
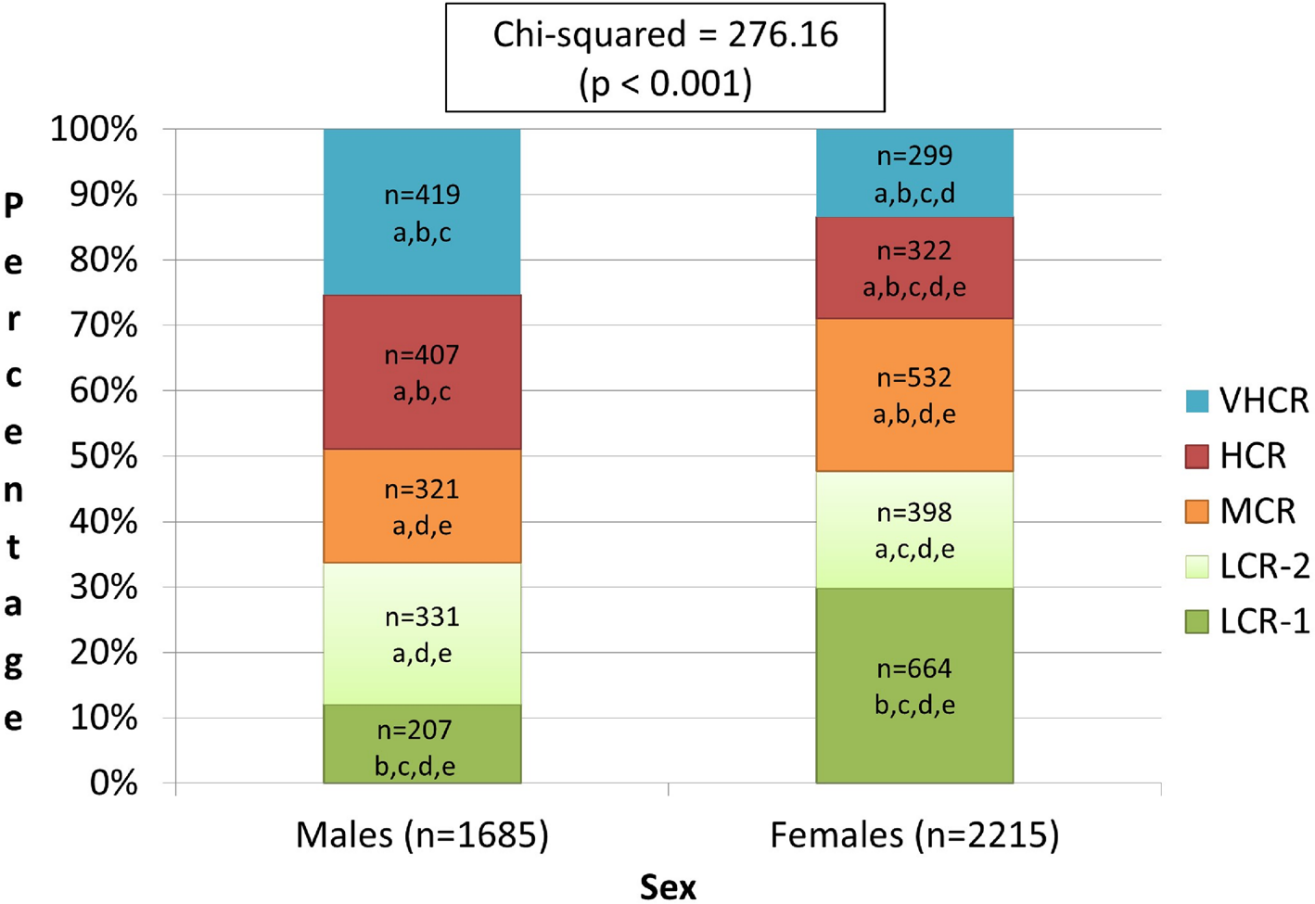
Cardiovascular risk clusters’ distribution based on sex (*n* = 3,900). a, Statisticaly significantly different from LCR-1 (*p* < 0.05, 2-tailed). b. Statisticaly significantly different from LCR-2 (*p* < 0.05, 2-tailed). c. Statisticaly significantly different from MCR (*p* < 0.05, 2-tailed). d. Statisticaly significantly different from VHCR (*p* < 0.05, 2-tailed). Abbreviations: VHCR, very high cardiovascular risk; HCR, high cardiovascular risk; MCR, moderate cardiovascular risk; LCR-2, low cardiovascular risk (group 2); LCR-1, low cardiovascular risk (group 1)

Among males, in comperason to the low and moderate risk custers (clusters 1 through 3), statsiticaly significantly greater prevalence was found in clusters 4 (high risk, 24.86% of the males) and 5 (very high risk; 24.50% of the males). In contrast, among females, the hight prevelance was in cluster 1 with 29.97% of the females belonging to this cluster (see Fig. 1).

**Fig. 2:**
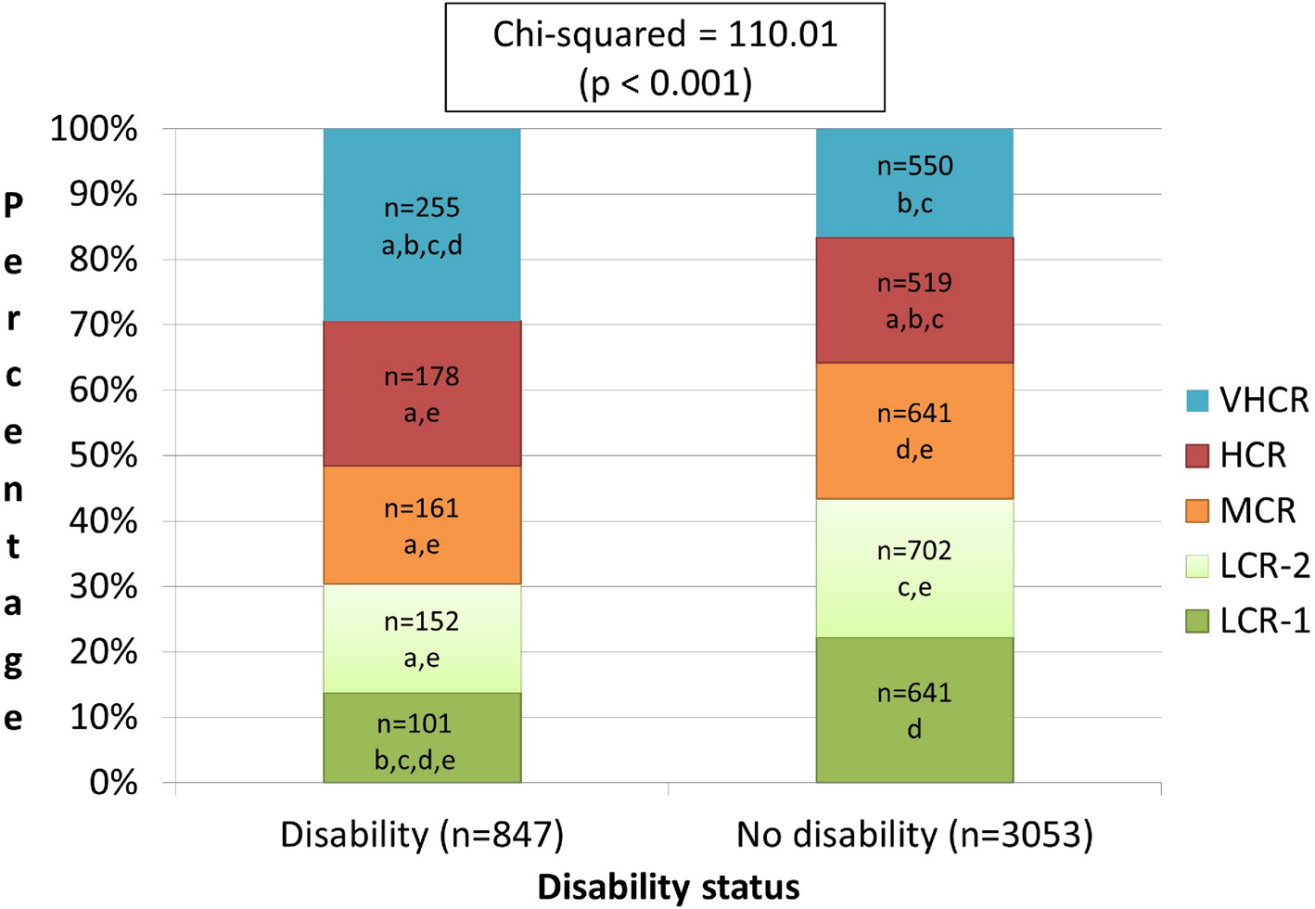
Cardiovascular risk clusters’ distribution based on disability status (*n* = 3,900). a, Statisticaly significantly different from LCR-1 (*p* < 0.05, 2-tailed). b. Statisticaly significantly different from LCR-2 (*p* < 0.05, 2-tailed). c. Statisticaly significantly different from MCR (*p* < 0.05, 2-tailed). d. Statisticaly significantly different from VHCR (*p* < 0.05, 2-tailed). e. Statisticaly significantly different from VHCR (*p* < 0.05, 2-tailed). Abbreviations: VHCR, very high cardiovascular risk; HCR, high cardiovascular risk; MCR, moderate cardiovascular risk; LCR-2, low cardiovascular risk (group 1); LCR-1, low cardiovascular risk (group 2)

In the disability goup the cluster with the hight prevelance was cluster 5 (very high risk; 30.10% of particpants with disability). In the non-disabeled group the most prevelant cluster was high risk cluster (22.99% of the group; see Fig. 2).

#### Prevalence of the CVDs risks among the various clusters

**Fig. 3:**
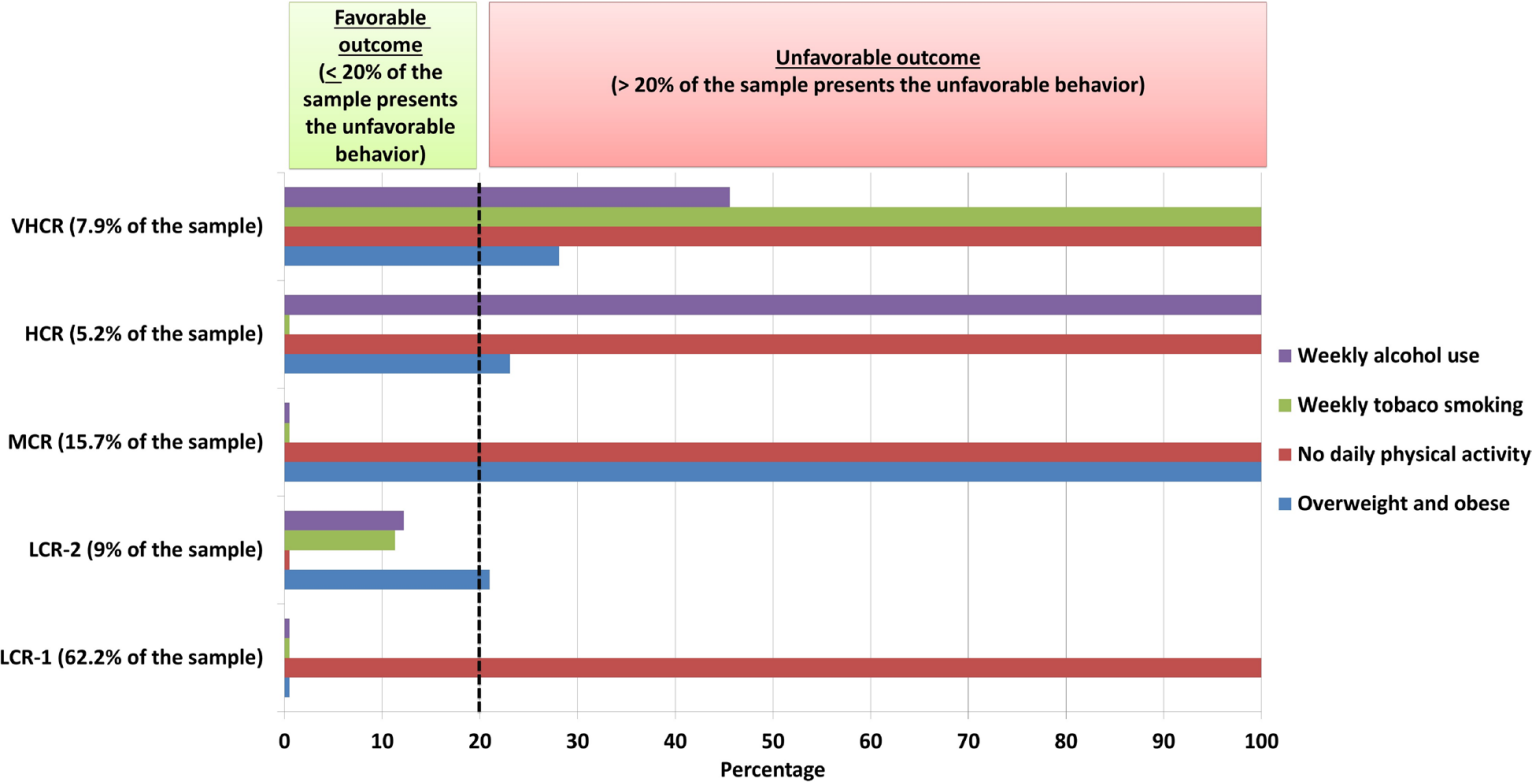
Within cluster percentage of favorable lifestyle behavior (*n* = 3,900). The vertical dashed black line represents the cut-off for favorable lifestyle (< 20% of the sample presented unfavorable lifestyle behavior). Abbreviations: VHCR, very high cardiovascular risk; HCR, high cardiovascular risk; MCR, moderate cardiovascular risk; LCR-2, low cardiovascular risk (group 2); LCR-1, low cardiovascular risk (group 1)

Adolescents in cluster 1 (low risk) did not present any risk behaviours for CVDs, except for not engaging sufficiently in daily physical activity (100% of the cluster). Similarly, those in cluster 2 (low risk-group 2) also presented only one risk factor: overweight and obesity (21.1% of the cluster). Lifestyle risks for CVDs in cluster 3 were overweight and obesity (100% of the cluster), as well as low level of physical activity (100% of the cluster). In cluster 4, overweight and obesity (23.2% of the cluster), low levels of physical activity (100% of the cluster), and alcohol use (100% of the cluster) were prevalent. Finally, in cluster 5, unfavourable behaviours of all four lifestyle behaviours were demonstrated (Table 3; Fig. 3).

## Discussion

The aim of the present study was to explore the existence of risks the development of CVDs among adolescents with disabilities. Bad habits and inadequate lifestyle characteristics, such as unhealthy diet, physical inactivity, tobacco smoking, and alcohol usage among adolescents, are related to the development of CVDs. The study was based on data from Israeli adolescents aged 11 to 19 years, collected from the 2018/2019 HBSC study.

We found that less than a quarter (21.1%) of the study participants were overweight or obese, only a minority (9%) of them participated in daily MVPA, as recommended, and most did not smoke or drink alcohol regularly [31, 39]

A total of 21.1% of the current study’s participants were disabled. A statistically significant higher percent of adolescents from in the disabled group was obese compared to those in the non-disabled group (25.8% vs.19.91%, respectively, *p* < 0.05). Similar findings have been recorded in other studies. Rimmer et al. conducted an online survey among 662 parents of adolescents aged 12 to 18 years with disabilities and compared their reports to the 2007 Youth Risk Behavior Survey data. Adolescents with disabilities demonstrated a higher prevalence of obesity compared to those who completed the Youth Risk Behavior Survey (17.5% vs. 13.0%, p = 0.04)) [32]. This could be because Rimmer’s study relied upon parent reports. In contrast, in the HBSC study, the students report their height and weight. Although it may be seen that parents would have better accuracy of the height and weight of their child, Rimmer’s study had fewer participants in the study and was recruited selectively rather than a larger sample in the HBSC study.

Our findings reveal that tobacco smoking and alcohol use were statsticaly significantly higher among the disabled group than the non-disabled group. Similarly, Janeković et al. found that adolescents with physical disability drink alcohol more often and with different motives than adolescents with no disability. However, Janeković et al. found no statistically significant difference between the tested groups regarding the prevalence, frequency, quantity, or motives for smoking [40]. Nagarajan et al. compared tobacco use among adolescents with physical disabilities to those without physical disabilities by utilizing data from six published cross-sectional surveys. They found that adolescents with physical disabilities were statsiticaly significantly more likely to use tobacco than adolescents without disabilities (29.7% vs. 23.3%, respectively) [41].

An additional study compared 319 adolescents with physical disabilities to 7,020 adolescents without physical disabilities in USA, which aimed to investigate age-related differences in health risk behaviours (including smoking and alcohol use). Significant age-related differences were found for having tried smoking, tasting an alcoholic drink, and being drunk. Most of these health risk behaviours were detected among the 15–16 years old with physical disabilities; the rate was similar to that reported among the 11–12 years old in the general population. Yet, an analysis of the associations between disability status and health risk behaviours (excluding age and sex) demonstrated that the disability was associated with a lower likelihood of health risk behaviours [42].

We found no statistically significant differences between the disabled vs. non-disabled groups regarding physical activity. This could be because low levels of physical activity were reported by all study participants, whether they were disabled or not [43]. [39]. Individuals with disabilities often experience impairments in their motor skills, including issues with balance, perceptual issues, hand–eye coordination, low muscular tone, and weakened physical posture, all of which can significantly contribute to their reduced physical activity [43, 44]. A study by Rowland and colleagues? found that lower extremity muscle strength was correlated with functional mobility in youth with cerebral palsy, Down syndrome, Duchenne muscular dystrophy and spina bifida [45]. Justin et al. found that adolescents with severe visual impairments participated in fewer moderate to vigorous physical activity days per week [46]. Thus, the low level of physical activity observed in our study could be explained by the fact that adolescents with disabilities have difficulties conducting physical activity due to the physical complications caused by their impaired pathological condition, whether cognitive, development or physical related.

Individuals in cluster 2 (low risk – group 2) were statistically significantly younger than the other four clusters. Most of the males were from the high and very high-risk groups (clusters 4 and 5), whereas most females were from the first low and moderate risk groups (clusters 1 and 3). This finding is consistent with data published by Musa et al., which revealed that girls presented a lower risk profile in developing CVDs than boys [47]. Alternatively, Thangiah et al., who conducted a study among 1320 Malaysian adolescents, found that female adolescents presented a higher risk for CVD [48].

We also found that the socioeconomic status of those presenting a very high CVD risk (cluster 5) was statisticaly significantly lower than those from the first and second low-risk groups (clusters 1 and 2) or from the high-risk group (cluster 4). Low socioeconomic status during childhood was found to be associated with increased insulin resistance during adolescence, which is known to be a risk factor in CVD development [49]. Data from the National Longitudinal Study of Adolescent to Adult Health (*N* = 14,493), which followed US adolescents through early adulthood, found that some low socioeconomic status elements (e.g., health behaviors, financial stress, lack of medical/dental care, educational attainment) that were present during adolescence statisticaly significantly predicted higher risk for CVDs in adulthood [50]. Seo et al. conducted a prospective longitudinal cohort study that found that low socioeconomic status was a significant risk factor for developing CVDs [51].

When examining the disabled as compared to the non-disabled group, the two-step cluster analysis for the risk of developing CVDs demonstrated that approximately 50% of youth with disability were at high and very high risk (clusters 4 and 5), approximately 20% and 30% of the clusters, respectively. On the other hand, most subjects without a disability were at the first low and moderate groups (clusters 1 and 3). The high rate of obesity, tobacco smoking, alcohol use, and low levels of physical activity among particpants with disability could cause the finding that most were at high and very high risk of developing CVDs.

Thus, it seems that to decrease the risk of developing CVDs among adolescents with disabilities, adapted and adjusted intervention plans and addressing the increasing rates of inappropriate lifestyle habits associated with the CVDs risks are desperately needed. Many previous studies have presented various intervention plans that target adolescent with disabilities, many of which aim to encourage weight loss and increase the rate of physical activity. Yu et al. examined the effectiveness of a 9-month school-based adapted physical activity program for reducing weight among 61 adolescents with intellectual disabilities. A significant post-intervention difference was found between the two groups, which demonstrated that a reduced BMI was found in the intervention group. At the same time, an increase in BMI was observed in the control group [52].

Unfortunately, health promotion intervention plans for adolescents with physical disabilities are relatively undeveloped, unlike those that target their non-disabled peers. In February 2016, a Canadian multi-stakeholder workshop that focused on obesity and health in children with physical disabilities published a white paper that recommended investing extensive efforts in studying weight-related topics in children with physical disabilities and accordingly developing evidence-based obesity prevention and treatment approaches [53]. Matizanadzo et al., conducted a systematic review aimed analyse the status of obesity interventions among children and youth with physical disability. The results, collected from 7 studies, showed no significant reduction in BMI or increase in obesity prevention knowledge. The authors concluded that obesity intervention plans for adolescents with physical disabilities are poorly designed and are conveyed, adjusted, and implanted improperly [54].

There is also a need for tobacco and alcohol use cessation programs for adolescents with disabilities. A study conducted by Pomeranz et al. revealed that community-based participatory research methods are needed to develop tobacco cessation programs for persons with disability [55]. Senders et al., who compared the prevalence of tobacco use among high school students with at least one disability to those without disability, suggested that tobacco prevention and reduction efforts should include adolescents with disabilities. The authors recommended that a disabled current or former smoker be involved in the delivery of the intervention plan. The plans should be tailored according to the different disabilities (e.g., visual, hearing, cognitive, mobility) [56].

A systematic literature review aimed to investigate the effects of school-based interventions or prevention programs directed at the reduction of alcohol, tobacco, and drug use in young adolescents with disabilities or physical impairments was conducted by Triantafyllou et al. Data from five studies demonstrated that although the interventions enhanced the participants’ knowledge about the risks of alcohol, tobacco, and drug usage, no change in the adolescents’ motives to use substances or to decrease their current substance use was observed. Due to the finding from the screened studies that 6% of the participants had smoked for the first time and 15% had consumed alcohol when they were 10 years or younger, it was recommended that the prevention efforts should start before the age of 12 [57].

This study had several limitations. First, this was a cross-sectional study; it only provides an association and not causation. Second, the application of convenience sampling and recalled method to collect data may elevate the risk of selection bias and recall bias, respectively. Third, it would be beneficial to assess whether previous studies have evaluated the correlation between such questionnaires in this age group with clinical cardiovascular outcomes (such as myocardial infarction, heart failure, etc.), rather than solely with cardiovascular risk factors. Although we adjusted all the potential risk factors in the binary logistic regression, there is also a possibility that there were residual confounders. However, because all indicators have been dichotomized, slight exaggerations and understatements should not play a role.

This study also has strengths. For example, as far as we know the study addressed, for the first time, the cluster incidence of CVD risk factors among disabled adolescence in Israel. For Israel’s public health regarding the adolescent disability population, it may be beneficial to create a policy that supports the development and implement CVD prevention programs. As the research demonstrated, classifying profiles of moderate- and high-risk disabled adolescents in Israel is crucial, It is well known that the associates between lifestyle bad behaviors, such as smoking, excessive alcohol intake, and CVD can be changed. Lifestyle interventions regarding those bad behaviors are highly recommended.

It is well acknowledged that the connections between lifestyle behaviors, such as physical inactivity, unhealthy eating, smoking, and excessive alcohol consumption, and CVD can be modified. Much change can be initiated through lifestyle interventions although some prescriptions are available for adolescents to use [49].

## Conclusion

The data from the current study demonstrate that adolscents with disability were found as having a higher risk of developing CVDs than their non-disabled peers. This tendency dervies directly from high rate of risk factors such as obesity, low physical activity rates, smoking, and alcohol use, which are found in average prevalent among this population. These factors are also found in the peer group of non-disabled subejects, but the level of presense does not lead significantly to development of CVDs cases.

Therefore, intervention programs are tailored to the needs of adolescents with disbility. Programs should be based on lifestyle habit change and promoting healthy living. They should be delivered and assimilated among the family, community, and school. Pediatric physical therapists, skilled educators and teachers, and appropriate resources, equipment, and facilities should be provided. Such programs could improve the well-being and health of disabled adolescents.

## Data Availability

The study’s results are backed by available data. Nonetheless, the data used in this study were obtained through licensing and are not publicly accessible due to restrictions. Access to the data may be granted upon reasonable request and with the consent of the Israel HBSC Program.

## Appendix

**Table 4:** Population descriptive statistics, missing data group vs. study group

Variable		Missing data group (*n* = 507)	Research group (*n* = 3900)	t-statistic (*p* value) OR Chi-squared (*p* value)
		**Mean (SD)** **[range]** **OR** **N (%)**	**Mean (SD)** **[range]** **OR** **N (%)**	
Age, years: mean (SD) [range]		13.63 (2.12) [11.00–18.00]	14.52 (2.07) [11.00–19.00]	-9.02 (< 0.001)
Sex, n (%)	Male	219 (43.19)	1685 (43.20)	0.001 (0.99)
	Female	288 (56.80)	2215 (56.79)	
Socioeconomic status (Family Affluence Scale), mean (SD) [range]^a^		8.08 (2.69) [0–17]	8.48 (2.51) [0–17]	-3.39 (0.001)
Disabilities, N (%)	No disabilities	393 (77.51)	3053 (78.28)	
	Disabilities	114 (22.48)	847 (21.71)	
	Seeing difficulties	49 (9.7)	390 (10.0)	0.04 (0.83)
	Hearing difficulties	54 (10.6)	382 (9.8)	0.32 (0.57)
	Walking difficulties	52 (10.2)	351 (9.0)	0.77 (0.37)
	Physiological disabilities	80 (15.8)	605 (15.5)	0.03 (0.86)
	Remembering difficulties	56 (11.1)	386 (9.9)	0.71 (0.39)
	Learning difficulties	55 (10.9)	406 (10.4)	0.09 (0.92)
	Concentrating difficulties	51 (10.0)	374 (9.6)	0.02 (0.96)
	Cognitive disabilities	80 (15.8)	601 (15.4)	0.01 (0.97)

*Abbreviation*: *SD* Standard deviation

^a^The Family Affluence Scale is a 6-item questionnaire. For the purpose of this study, a total score was calculated. Total score ranges from 0 (lowest) to 17 (highest)
